# Assessment of coastal river water quality in Bangladesh: Implications for drinking and irrigation purposes

**DOI:** 10.1371/journal.pone.0300878

**Published:** 2024-04-18

**Authors:** Md. Ripaj Uddin, Mayeen Uddin Khandaker, Shamim Ahmed, Md Jainal Abedin, Syed Md. Minhaz Hossain, Muhammad Abdullah Al Mansur, Shakila Akter, Md. Ahedul Akbor, AHM Shofiul Islam Molla Jamal, Mohammed M. Rahman, Mohsin Kazi, Md. Abu Bakar Siddique, Abubakr M. Idris

**Affiliations:** 1 Institute of National Analytical Research and Service (INARS), Bangladesh Council of Scientific and Industrial Research (BCSIR), Dhanmondi, Dhaka, Bangladesh; 2 Institute of Mining, Mineralogy and Metallurgy (IMMM), BCSIR, Joypurhat, Bangladesh; 3 Applied Physics and Radiation Technologies Group, CCDCU, School of Engineering and Technology, Sunway University, Subang Jaya, Selangor, Malaysia; 4 Faculty of Graduate Studies, Daffodil International University, Daffodil Smart City, Birulia, Savar, Dhaka, Bangladesh; 5 Faculty of Public Health, Thammasat University, Bangkok, Thailand; 6 Department of Computer Science and Engineering, Premier University, Chattogram, Bangladesh; 7 Department of Chemistry, Faculty of Science, King Abdulaziz University, Jeddah, Saudi Arabia; 8 Department of Pharmaceutics, College of Pharmacy, King Saud University, Riyadh, KSA; 9 Department of Chemistry, King Khalid University, College of Science, Abha, Saudi Arabia; CIFRI: Central Inland Fisheries Research Institute, INDIA

## Abstract

Saltwater intrusion in the coastal areas of Bangladesh is a prevalent phenomenon. However, it is not conducive to activities such as irrigation, navigation, fish spawning and shelter, and industrial usage. The present study analyzed 45 water samples collected from 15 locations in coastal areas during three seasons: monsoon, pre-monsoon, and post-monsoon. The aim was to comprehend the seasonal variation in physicochemical parameters, including water temperature, pH, electrical conductivity (EC), salinity, total dissolved solids (TDS), hardness, and concentrations of Na^+^, K^+^, Mg^2+^, Ca^2+^, Fe^2+^, HCO_3_^-^, PO_4_^3-^, SO_4_^2-^, and Cl^-^. Additionally, parameters essential for agriculture, such as soluble sodium percentage (SSP), sodium absorption ratio (SAR), magnesium absorption ratio (MAR), residual sodium carbonate (RSC), Kelly’s ratio (KR), and permeability index (PI), were examined. Their respective values were found to be 63%, 16.83 mg/L, 34.92 mg/L, 145.44 mg/L, 1.28 mg/L, and 89.29%. The integrated water quality index was determined using entropy theory and principal component analysis (PCA). The resulting entropy water quality index (EWQI) and SAR of 49.56% and 63%, respectively, indicated that the samples are suitable for drinking but unsuitable for irrigation. These findings can assist policymakers in implementing the Bangladesh Deltaplan-2100, focusing on sustainable land management, fish cultivation, agricultural production, environmental preservation, water resource management, and environmental protection in the deltaic areas of Bangladesh. This research contributes to a deeper understanding of seasonal variations in the hydrochemistry and water quality of coastal rivers, aiding in the comprehension of salinity intrusion origins, mechanisms, and causes.

## 1. Introduction

Salinity refers to the saltiness or amount of salt dissolved in a water body. These salts include sodium chloride (NaCl), magnesium sulfate (MgSO_4_), potassium nitrate (KNO_3_), and sodium bicarbonate (NaHCO_3_), which dissolve in water to produce ions. The percentage of salt in various waters is categorized as freshwater (less than 0.5%), brackish (between 0.5 and 30%), saline (between 30 and 50%), and brine (more than 50%) [1]. Globally, salinity intrusion is a pressing environmental issue, affecting approximately 20 million people through soil and water in Bangladesh alone. The coastal belt of Bangladesh, covering 32% of the country and encompassing 19 districts [2], has seen a 26% increase in salinity over the last 35 years [3, 4]. The coast’s connection to major rivers through various water inlets and estuaries near the Bay of Bengal makes this bay a primary source of saline water. Cosmic factors, such as the positions and movements of the Earth and sun, indirectly influence environmental elements like temperature and sea-level rise, which can affect salinity. However, these processes occur at a very slow pace. Anthropogenic activities, including deforestation, inadequate water governance, industrial growth, and the extensive use of groundwater for domestic and agricultural purposes, are significant contributors to salinity intrusion. These human-induced actions, coupled with poor infrastructure maintenance and cross-boundary river policies, exacerbate the exchange of cations and anions in the salty aquifer, leading to increased salinity [5]. During the full moon, tidal pressures can easily weaken embankments, allowing saline water to enter the polders and causing salinity intrusion. This intrusion is exacerbated by factors such as tidal flow, tidal discharge, wind flow, oceanic pressure, sea-level rise, melting glaciers, and the expansion of oceans [6]. Bangladesh’s coastal areas are particularly vulnerable to disasters due to their geographic location and the tropical monsoon climate, which brings cyclones from May to November. Cyclones, originating in the Indian Ocean, contribute to increased water salinity in these regions. Between 1584 and 2021, 62 tropical cyclones have struck Bangladesh’s coastal belt, resulting in significant damage. Notable cyclones occurred in years such as 1584, 1699, 1767, 1822, 1831, 1847, 1876, 1958, 1960, 1962, 1963, 1965, 1966, 1970, 1974, 1991, 1994, 1995, 1997, 1998, and 2007. Specifically, in 1876, 1970, 1991, and 2007, cyclones reached maximum wind speeds of 222 km/h, 240 km/h, and 260 km/h, with surge heights of 13.6 m, 10.6 m, 8 m, and 12 m, respectively, illustrating the severe impact of these natural events on the region [7].

Global temperatures have increased significantly due to economic growth and greenhouse gas emissions, with an average rise of approximately 0.9°C to 1.2°C (equivalent to 1.6°F to 2.2°F) above pre-industrial levels [8, 9]. The Ganges-Brahmaputra-Meghna (GBM) river delta receives substantial freshwater inflow from the melting of Himalayan ice. Monitoring of the Mean Sea Level [10] shows that the global sea level has risen by 210 mm over the past 130 years, with the rate of increase accelerating in recent decades. Predictions suggest that by 2050, sea levels could rise by up to 1 meter along the Bangladesh coast, potentially displacing 5.73 million people [9]. In the region of Char Changa, an annual geological uplift of 0.018 mm has been observed [11]. Consequently, areas like Hatiya have faced regular tidal flooding and riverbank erosion, with a net sea level rise of 2.94 mm over 20 years. Specific locations such as Hiron Point, Char Changa, and Cox’s Bazaar have experienced sea level rises of 4 mm/year, 6 mm/year, and 7.8 mm/year, respectively. Bangladeshi rivers have deposited at least 2.4 billion tonnes of sediment annually for the past 5,000 to 7,000 years. This sediment accumulation has contributed to the formation of new charlands, although it also influences local sea level dynamics on riverbeds [12, 13]. Tidal ranges along India’s western and eastern (central coast) borders vary from 3 meters to 5 meters at the Meghna estuary mouth, where the average elevation above sea level is less than 3 meters [14]. In the catchment area, which includes canals, inlets, and rivers, salt levels are higher due to changes in fluvial morphology that decrease freshwater discharge and lead to shoreline waterlogging. The balance between groundwater aquifer recharge and outflow is crucial for preventing saltwater intrusion into freshwater systems. When groundwater aquifers deplete faster than they recharge, saltwater intrusion becomes a risk. The tropical monsoon brings high tides and salty water overflows along Bangladesh’s coastline, but moderate to high humidity and temperature result in significant rainfall between May and October, mitigating salinity effects. In 2001, the Huanghe Delta extracted 100 million m^3^ of groundwater for irrigation and aquaculture [15]. Strategies like those in the Mississippi Delta, which involve irrigating with upstream freshwater to prevent agricultural salinization, and the construction of canals and dikes in the Mekong Delta to supply freshwater irrigation, have paradoxically increased salinity. Brackish water from prawn cultivation has exacerbated soil and aquifer salinization in deltas such as Huanghe [16], Mekong, and GBM [17]. Freshwater inflow into deltas, affected by upstream water and land management, leads to salinization from upstream to downstream. Dams and water diversions on major rivers (e.g., GBM, Mekong, Rhine, and Meuse) [18], and consumptive agricultural water use significantly reduce inflow to deltas and distributaries globally. Water diversion within deltas, such as in Venezuela’s Orinoco delta where a distributary was dammed for hydroelectricity, poses challenges [19]. Additionally, the loss of sediment influx in deltas like the Mississippi has led to wetland subsidence, increased risk of storm surge flooding, and groundwater saline intrusion [20].

Surface water chemistry is essential for providing insights into water types, geochemical processes, and classifications based on hydrochemical benchmarks. By integrating surface water chemistry with geochemical characteristics, we can identify trends, environmental challenges, and enhance our understanding of water sources, geochemical processes, water quality, and susceptibility to contamination. The number and types of salts in water are critical factors affecting irrigation quality, with increased salinity, decreased permeability, and the presence of harmful ions significantly degrading water quality [21, 22]. Therefore, physicochemical properties are utilized to evaluate irrigation quality [23], employing replicative methods such as those developed by the US Salinity Laboratory [24] and the Wilcox Diagram [25]. These methods assess the suitability of water for irrigation purposes. Beyond these, Water Quality Indices (WQIs) are instrumental in evaluating the irrigation quality of water. However, relying solely on one indicator for assessing irrigation water’s validity can be misleading and result in poor assessment performance [21, 26]. Numerous studies have advocated for a water quality index that uses component-weighted scores to provide a more nuanced evaluation [26]. WQIs, incorporating factors like Sodium Percentage (Na%), Sodium Adsorption Ratio (SAR), Permeability Index (PI), Kelly’s Index (KI), and Residual Sodium Carbonate (RSC), are designed to meet comprehensive monitoring and assessment criteria by consolidating various factors into a singular measure. This aims to classify waters based on their potential uses, chemical composition, and physical properties to guide water allocation effectively. In Limpopo, South Africa [27], demonstrated that the IWQ is an effective tool for monitoring the Luvuvhu Catchment, with parameters such as Electrical Conductivity (EC), Na%, SAR, PI, and RSC providing reliable predictions of irrigation water quality [28, 29]. Additionally, the adoption of multivariate statistical methods, like Cluster Analysis (CA), which groups physicochemical properties based on the interactions between water’s chemical components, has gained popularity. Such geochemical and multivariate analyses are crucial for a comprehensive understanding of water quality and the processes affecting especially contaminated surface waters [30]. Principal Component Regression (PCR) is frequently used to predict hyperspectral responses in samples, offering valuable insights into the in situ canopy and broader environmental monitoring efforts [31, 32].

Prior research has not extensively evaluated the suitability of coastal waters of the GBM delta for irrigation and drinking purposes. This study aims to identify the spatiotemporal variations in salinity intrusion within the estuarine systems of the GBM delta, offering insights into how these changes impact water quality over time and space. Additionally, this study will determine the entropy-weighted water quality, a method that considers the variability and distribution of water quality parameters, to assess its suitability for drinking and irrigation purposes. By integrating these approaches, the study seeks to provide a comprehensive understanding of water quality dynamics in the GBM delta and inform sustainable water management practices.

## 2. Materials and methods

### 2.1 Study area

#### 2.1.1 Permission for sample collection

No permission was required to collect the water samples for this study. The samples were collected from the coastal area adjacent to the Bay of Bengal. As the sampling locations are not under any specific governance or private ownership, there was no requirement to obtain permission from individuals or institutions for sample collection.

#### 2.1.2 Geographical information of the sampling area

**Fig 1:**
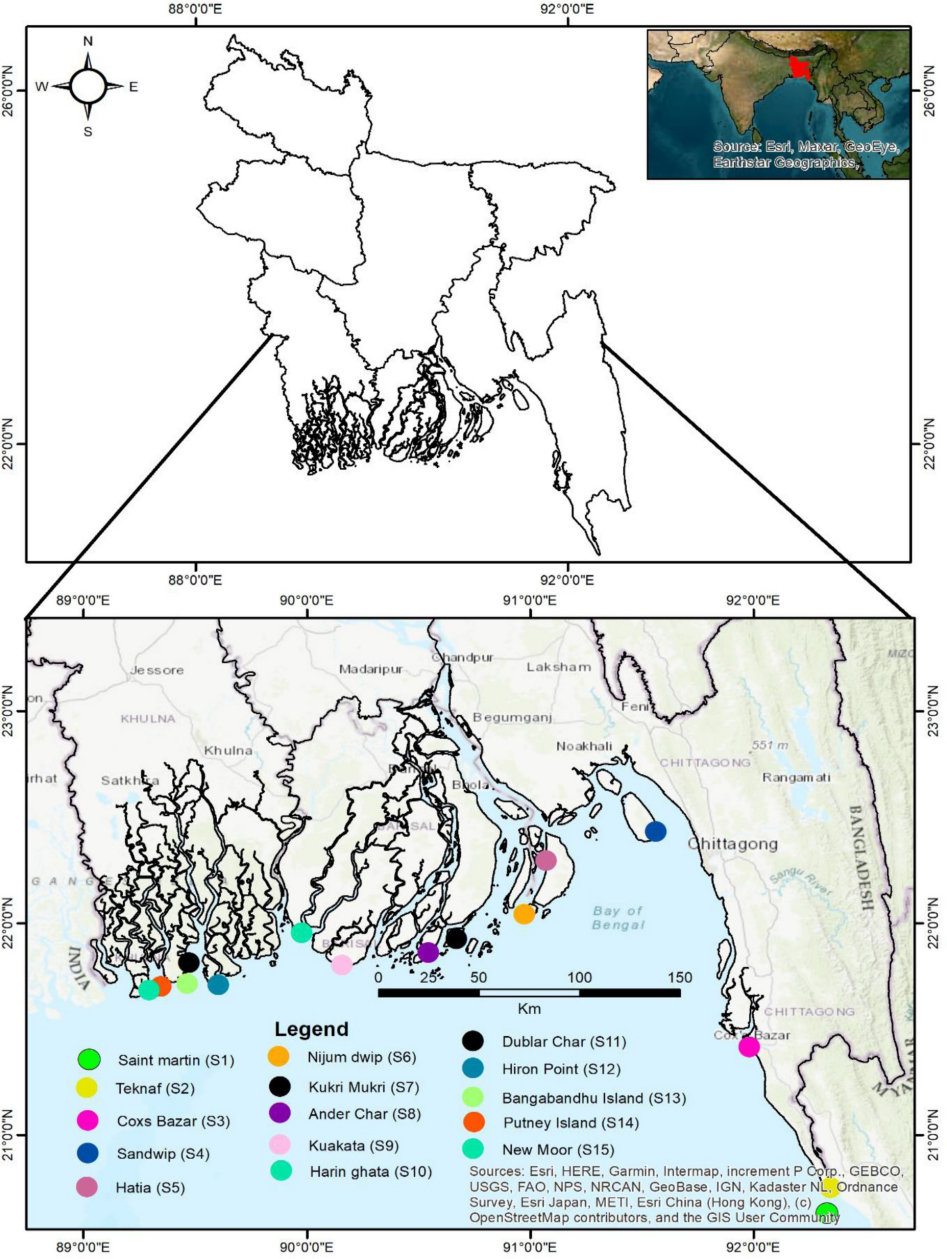
Sampling locations throughout the coastal belt of the Bay of Bengal (Southern region of Bangladesh).

Covering an area of 47,201 km^2^ [33], the coast of Bangladesh (Fig 1) is one of the most complex and dynamic deltas in the world [34]. The estuarine system of the GBM delta is classified into three parts: the Western Estuarine System (WES), Eastern Estuarine System (EES), and Central Estuarine System (CES). This classification is based on their hydromorphological characteristics, which are influenced by distinct geological formations and seasonal variations [35].

The three main rivers, Ganges, Brahmaputra, and Meghna, carry approximately 90% of the freshwater discharges from upstream, mixing with the saline water of the sea in the estuaries. The region features relatively flat geography, contrasting with the hilly terrain of the Chittagong region that slopes gently toward the Bay of Bengal [4], resembling an inverse funnel. The active lower Meghna estuary, including rivers like Tertulia, Lohalia, Feni, Halda, Karnafuli, Sangu, and Matamuhuri, drains through the combined flow of the GBM river systems. It is predominantly influenced by the Eastern Estuarine System (EES) region and flows into the northeastern corner of the ocean through this estuary. The Buriswar, Bishkhali, and Baleshwar estuaries, part of the Central Estuarine System (CES) coasts, consist mainly of plain land [35]. They are interconnected with the Tetulia estuary and divert GBM river channels. The world’s largest mangrove forest, the Sundarbans, located in the CES region, is ecologically very sensitive to salinity intrusion [36]. The underlying alluvial aquifer systems, hydraulically connected to the complex network of rivers and channels, are influenced by significant tidal activity and sea-level fluctuations, which impact water availability during low tide (1 m) and high tide (3.1 m) daily. The coastal aquifer’s lithology comprises thick deposits of silty clay, swampy peat soil, and coarse to fine-grained sandstone, covered with deltaic sediments and alluvial deposits, with the region’s aquifer dynamics strongly affected by active neotectonism. As shown in Fig 1 and Table 1, a total of 45 water samples were collected across the coastal belt areas, located between 92°19’39.3"E and 89°17’34.6"E longitude and 20°37’50.6"N and 21°40’57.8"N latitude.

**Table 1:** Sampling site (longitude and latitude) in the coastal belt.

ID	Location	Longitude	Latitude
S1	Saint martin	20°37’50.6"N	92°19’39.3"E
S2	Teknaf	20°45’02.5"N	92°20’43.4"E
S3	Cox Bazar	21°24’52.3"N	91°58’55.3"E
S4	Sandwip	22°25’49.6"N	91°33’42.7"E
S5	Hatia	22°17’36.3"N	91°04’15.1"E
S6	Nijum dwip	22°02’29.9"N	90°58’28.2"E
S7	Kukri Mukri	21°55’32.3"N	90°40’12.9"E
S8	Ander Char	21°51’38.9"N	90°32’35.8"E
S9	Kuakata	21°48’04.3"N	90°09’28.1"E
S10	Harin ghata	21°57’15.0"N	89°58’37.6"E
S11	Dublar Char	21°42’29.5"N	89°36’13.5"E
S12	Hiron point	21°48’45.5"N	89°28’23.0"E
S13	Bangabandhu Island	21°42’50.6"N	89°27’51.9"E
S14	Putney Island	21°42’00.9"N	89°20’48.6"E
S15	New Moor	21°40’57.8"N	89°17’34.6"E

### 2.2. Hydromorphological and hydrogeological settings

**Fig 2:**
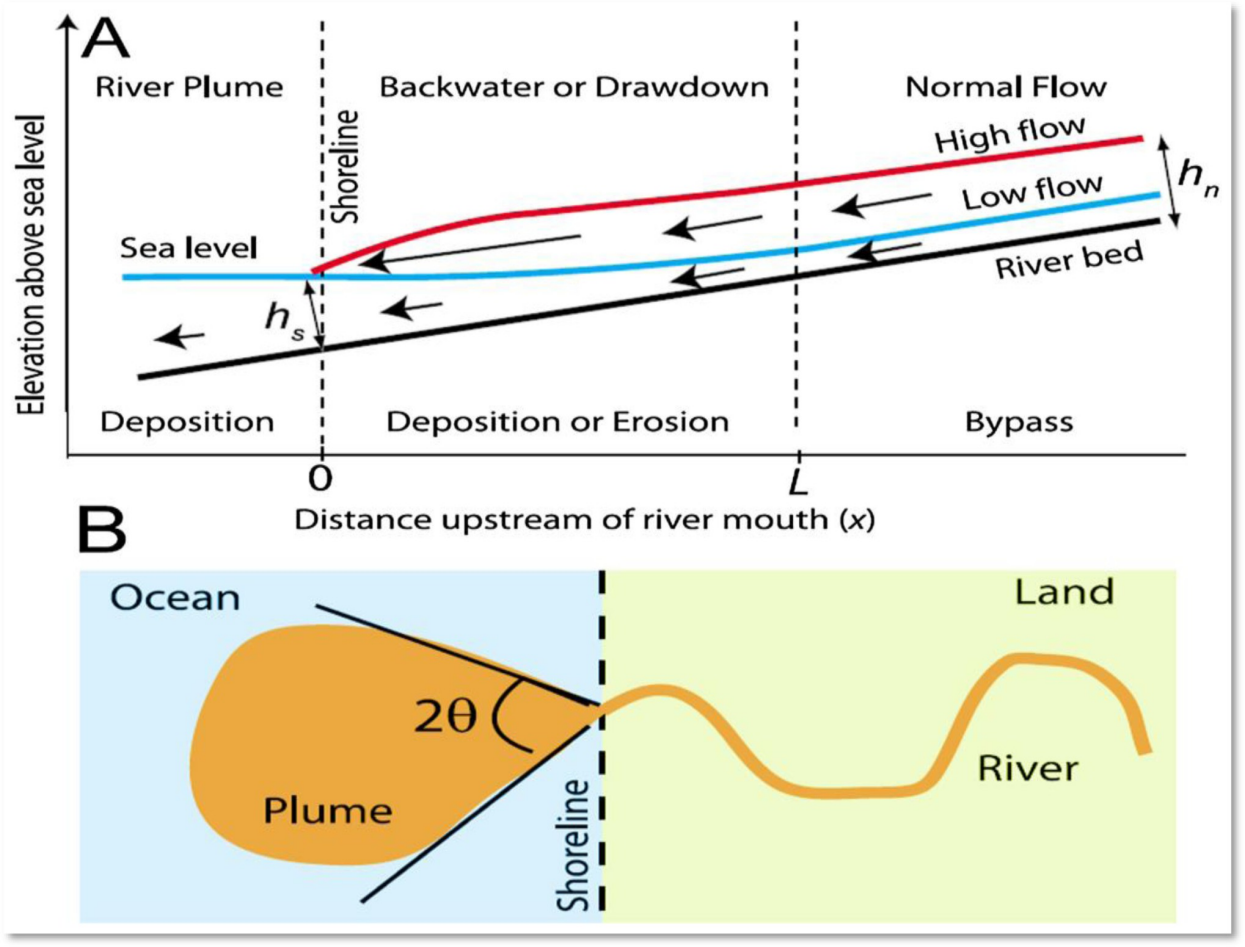
Tidal river dynamics and implementation for deltas; (A) cross-section and (B) plan view [39, 40].

The coastal region of the GMB Rivers forms the Bengal Foredeep Basin, with its eastern part known as the Bengal Flood Plain [37, 38]. This area is subject to various physical and meteorological phenomena, including storm surges, tropical cyclones, southwest monsoon winds, tides, and tidal flows from the Bay of Bengal. These factors contribute to the mixing of chemical compositions in water through ion exchange, with backwater effects from the sea to the river depicted in Fig 2(A). The Muhuri project, located at the Feni river mouth within the Central Estuarine System (CES), introduces a groundwater-based irrigation scheme that interacts with the saline intrusion affecting coastal belt water and aquifers, impacting agricultural practices such as shrimp farming, which has led to increased soil and water salinity. River systems flow into the ocean in three distinct manners, illustrated in Fig 2(A). High flow occurs when the coastal line (hs) exceeds the depth (hn), leading to erosion and increased flow velocity. In the transition area, the depth remains significant along the channel network, facilitating the observation of an offshore river plume, with mean sea level variations influencing river depth. Conversely, when the shoreline depth (hs) surpasses the typical flow depth (hn), low flow and backwater effects emerge, altering the flow area as indicated by the length of the arrows in the figure. These dynamics cause water surface elevation near the river mouth, especially due to transverse plume spreading [39]. Seasonally, from June to October, Chandpur experiences a discharge of 2.5 million cusecs, while the estuary discharges up to 4 million cusecs [40]. In contrast, winter water flow reduces to nearly 1/8 of this volume due to the river’s slowness and the broad estuary. During the rainy season, water flow can exceed 5 million cusecs, highlighting the significant seasonal variability in the region’s hydrology [41].

**Fig 3:**
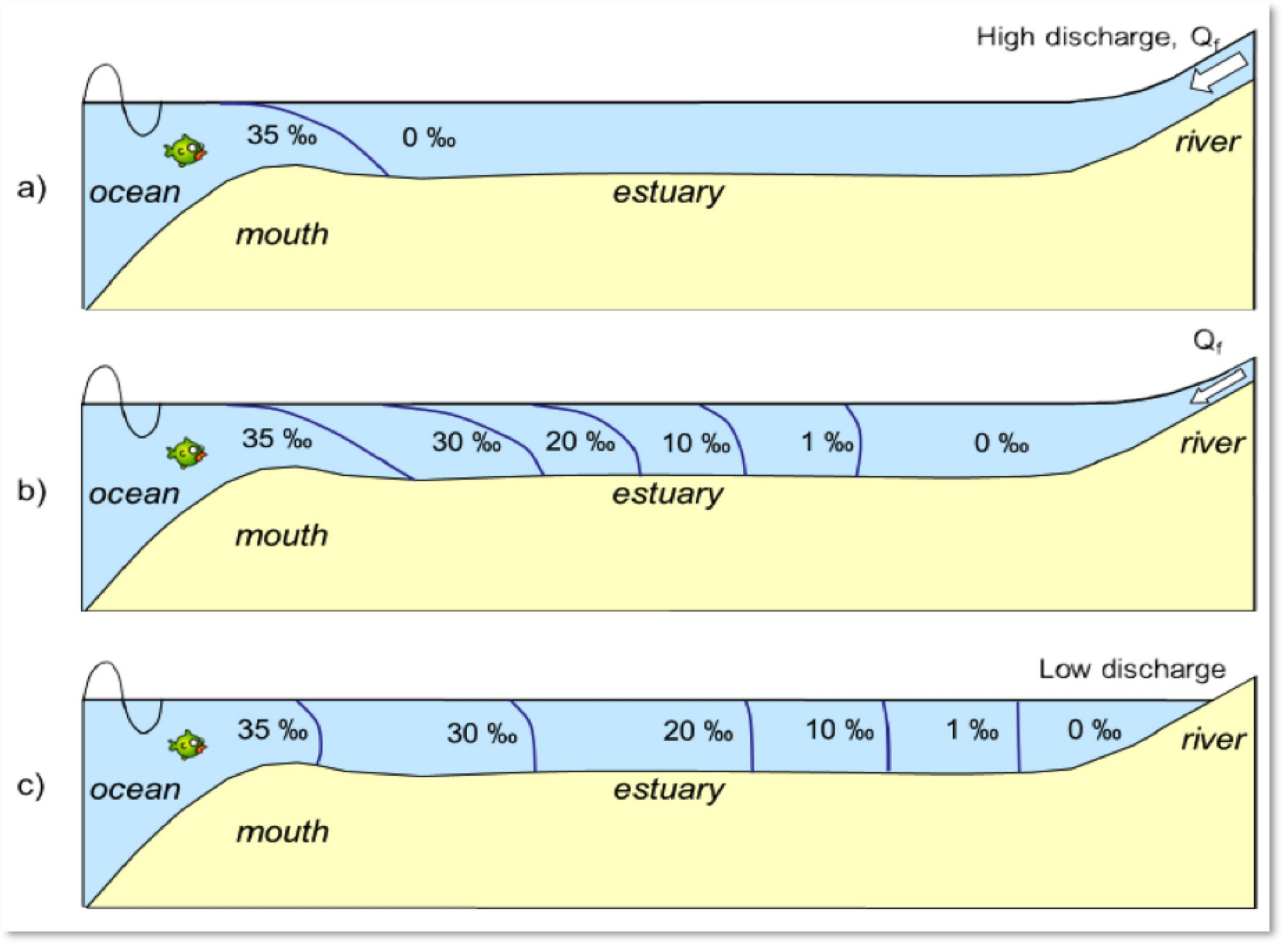
**(a).** A cross-laminated estuary, **(b)** a partially laminated estuary, and **(c)** a well-mixed laminated estuary [41].

Salt intrusion occurs when saltwater, which is 2–3% heavier than freshwater, extends over tens of kilometers. In this dynamic, freshwater floats on top of saltwater, leading to a stratification effect. Fig 2 illustrates the process and mechanism behind salt intrusion. The mixing of fresh and saltwater, along with counterpressure from freshwater inflow, serves to reduce salinity further upstream. Salt intrusion influences river discharges, sea levels, and the morphology of estuaries. At the juncture where the river meets the sea, saltwater can penetrate the estuary, as shown in Fig 3(A). Technologies designed to halt salt intrusion operate effectively under normal conditions in river estuaries. However, due to the construction of upstream barrages, the rate of river discharge decreases, enabling saltwater to move inland more rapidly, as depicted in Fig 3(B) and 3(C). In Bangladesh, the discharge rate of the Ganges River at the Hardinge Bridge dropped to 150 m^3^/s in 1995 from a previous 2,000 m^3^/s, as referenced in [33, 42]. Following the construction of the Farakka Barrage, there was a significant alteration in the hydrology of the Ganges. The Ganges-Brahmaputra-Meghna (GBM) river system could accommodate saltwater navigation if fish ladders and ships are able to pass through sluice gates.

### 2.3. Sample collection, preservation, preparation, and analysis

During the pre-monsoon, monsoon, and post-monsoon seasons of 2020, water samples were collected from various coastal regions of Bangladesh. As indicated in Table 1 and Fig 1, a total of 45 water samples were collected across the coastal belt area. Samples S1 through S15 were collected before the monsoon season, S16 through S30 during the monsoon season, and S31 through S45 after the monsoon season. The sampling locations were selected at the river system’s low tide. Before storage, each bottle was accurately labeled with the date, location, and time of collection and then stored in a refrigerator at 4°C in the dark. The collected samples were analyzed using the Laboratory Quality Management System (LQMS) and standard methods, such as those outlined in the American Public Health Association (APHA) guidelines (2017) [43]. Specific physicochemical parameters, including water temperature, total dissolved solids (TDS), turbidity, salinity, and electrical conductivity (EC), were measured. Freshwater samples’ parameters were measured on-site with a calibrated portable multimeter (Model: SENSION™156, HACH, USA), while pH and dissolved oxygen (DO) levels were determined using pH meters (Model: HannaHI-255, USA) and DO meters (Model: YK), respectively. Sodium and potassium contents were measured using a flame photometer (Model: PFP7, Jenway, UK). Bicarbonate concentration was determined with 0.2 N HCl, using phenolphthalein as the indicator. Total water hardness was calculated using a complexometric method (TH), expressed in milligrams per liter (mg/L) of calcium carbonate. The hardness was calculated using Eq (1).


$$Hardness\;as\;{CaCO}_{3}=(A\times B\times 100\times 1000)/(sample\;volume\;in\;mL)$$ (1)

Where, A = Volume of titrant (EDTA) is used in mL and B = Molarity of titrant.

An atomic absorption spectrophotometer (Model: AAS240FS, Varian, Australia) was utilized to determine the concentrations of other metallic ions (cations), such as Ca^*2+*^, Mg^*2+*^, and Fe^*2+*^. The concentrations of anions such as Cl^*-*^, NO_*3*_^*-*^ and SO_*4*_^*2-*^ were analysed by ion chromatography (Model: HIC-10A super, Shimadzu, Japan), while dissolving 0.2856 g of sodium bicarbonate (1.7 mM) and 0.3816 g of sodium carbonate (1.8 mM) in water and diluting to 2 L with deionized water. PO_*4*_^*3-*^ was investigated using an UV‒Vis spectrophotometer (model: UV-1650PC, Shimad Zu, Japan).

### 2.4. Quality control for accurate analysis

The study’s water samples were analyzed comprehensively in accordance with the ISO/IEC 17025:2017 standards. All calibrated glassware, including pipettes, burettes, volumetric flasks, and beakers, was thoroughly washed with deionized water and then rinsed with 2% (v/v) HNO_3_. An electric digital balance (HR-200, Max 210 g, d = 0.1, A&D Company Limited, Japan; Model N92, D0001), which had been calibrated, was utilized for weighing the samples. Deionized water (Barnstead International, USA, Model-D7071, Resistance 18.2 MΩ·cm, Conductivity 0.2 μS/cm at room temperature), certified reference material (CRM) reagents (Sigma Aldrich, Germany), and CRM stock standards (concentration 100 ± 4 mg/L) were employed for preparing the samples and standards and for calibrating the instruments. Quality control and assurance measures, including spike recovery, independent standard verification, blank checks, and duplicate analyses, were conducted. The correctness and precision of the analytical data were verified through triplicate analyses using traceable CRM standards, samples, and reagents, and a quality control chart was maintained to monitor these analyses. The concentrations of various metals and anions were determined using atomic absorption spectrophotometry (AAS) and ion chromatography (IC) methods, employing calibration standards that were diluted from stock standards. The detection limits for heavy elements such as Na, Mg, K, Ca, and Fe were 1.0, 0.1, 1.0, 0.1, and 0.2 mg/L, respectively. For metal analysis, the digestion process involved taking a 100 mL water sample, adding 2 mL of concentrated HNO_3_ (67–70%), and then evaporating the mixture to reduce the volume to 40 mL. The sample was subsequently filtered using filter paper (Whatman 42 μm) after being diluted to the appropriate volume with distilled water [44–47].

### 2.5 Agricultural suitability analysis

The quality of water for irrigation purposes is determined by several key factors: Soluble Sodium Percentage (SSP), Sodium Adsorption Ratio (SAR), Kelly’s Ratio (KR), Magnesium Adsorption Ratio (MAR), Permeability Index (PI), and Residual Sodium Carbonate (RSC). These parameters are derived using specific formulas, and their results are presented in Table 2. The SSP was calculated using Eq (2), as provided by Todd (1980), with the concentration of all ions expressed in mg/L. The sodium hazard was assessed using the percentage of sodium (Na%) [48].


$$SSP\;or\;\%{Na}^{+}=\frac{{Na}^{+}+K^{+}}{{Ca}^{2+}+{Mg}^{2+}+{Na}^{+}+K^{+}}$$ (2)

The SAR was determined using Eq (3) by Richards (1954) [49].


$$SAR=\frac{{Na}^{+}}{\sqrt{\frac{{Ca}^{2+}+{Mg}^{2+}}{2}}}$$ (3)

Kelly’s ratio was determined via Kelly’s formula [50],

$$KR=\frac{{Na}^{+}}{{Ca}^{2+}+{Mg}^{2+}}$$ (4)

The MAR was determined using Eq (5) given by (Raghunath, 1987) [51],

$$MAR=\frac{{Mg}^{2+}\times 100}{{Ca}^{2+}+{Mg}^{2+}}$$ (5)

The P.I. was determined using Formula (6)

$$PI=\frac{({Na}^{+}+{{HCO}_{3}}^{-})\times 100}{{Ca}^{2+}+{Mg}^{2+}+{Na}^{+}}$$ (6)

The RSC was determined using Gupta’s formula [52]

$$RSC=({{CO}_{3}}^{2-}+{{HCO}_{3}}^{-})-({Ca}^{2+}+{Mg}^{2+})$$ (7)

**Table 2:** Suitability test of data for factor analysis of water samples.

Suitability test of data for factor analysis				
Cronbach’s Alpha depends on standardized parts	Sampling Adequacy evaluation by KMO test	Bartlett’s Test of Sphericity		
		Chi-Square (χ2)	D_f_	P value (Sig.)
0.81	0.83	948.99	171.0	0.0

### 2.6. Assessment and characterization of the water quality

The study includes a comparison of analyzed parameters with the permissible limits for drinking water quality set by various agencies, including the Bureau of Indian Standards (BIS) in 2012 [53], the U.S. Environmental Protection Agency (US EPA) in 2018 [54], the World Health Organization (WHO) in 2011 [55], and The Environment Conservation Rules (ECR) in 1997 [56]. Additionally, the entropy-based water quality index (EWQI) was calculated using consecutive footprints to facilitate proper evaluation [57]. The eigenvalue matrix (A) is revealed and estimated by Eq (8), where the entropy weight ’i’ (i = 1, 2,…, m) represents the number of river water samples, and ’j’ (j = 1, 2,…, n) represents the number of analyzed parameters.


$$A=\left(\begin{array}{ccc} a11 & a12\ldots & a1n\\ \begin{array}{c} a21\\ .\\ . \end{array} & \begin{array}{c} a22..\\ .\\ . \end{array}\ddots & \begin{array}{c} a2n\\ .\\ . \end{array}\\ am1 & am2\ldots & amn \end{array}\right)$$ (8)

Eqs (9, 10) are used to evaluate the efficiency and normalization of the analysed parameters. The eigenvalue matrix (A) was resolved into a standard grade matrix (B) for assessing the unique measurement units from various measurement units of the analysed parameters [58].


$$B=\left(\begin{array}{ccc} b11 & b12.. & b1n\\ \begin{array}{c} b21\\ .\\ . \end{array} & \begin{array}{c} b22..\\ .\\ . \end{array}\ddots & \begin{array}{c} b2n\\ .\\ . \end{array}\\ bm1 & bm2.. & bmn \end{array}\right)$$ (9)

$$Bij=\frac{Aij-(Aij)max}{(Aij)max-(Aij)min}$$ (10)

$$Cij=\frac{Bij}{\sum_{i=1}^{m}Bij}$$ (11)

$$ej=-\frac{1}{\ln\left(m\right)}\sum_{i=1}^{m}(Cij\times \ln\;Cij)$$ (12)

$$wj=\frac{1-ej}{\sum_{j=1}^{m}\left(1-ej\right)}$$ (13)

$$qj=\frac{Cj}{Sj}\times 100$$ (14)

Where qj = quality rating, Cj = concentration of each chemical parameter in each water sample, and Si = drinking water standard for each chemical parameter (BIS, 2012). By using Eqs (11–15), the EWQI can be calculated.


$$EWQI=\sum_{j=1}^{n}wj\times qj$$ (15)

### 2.7. Basic multivariate statistical analysis

**Fig 4:**
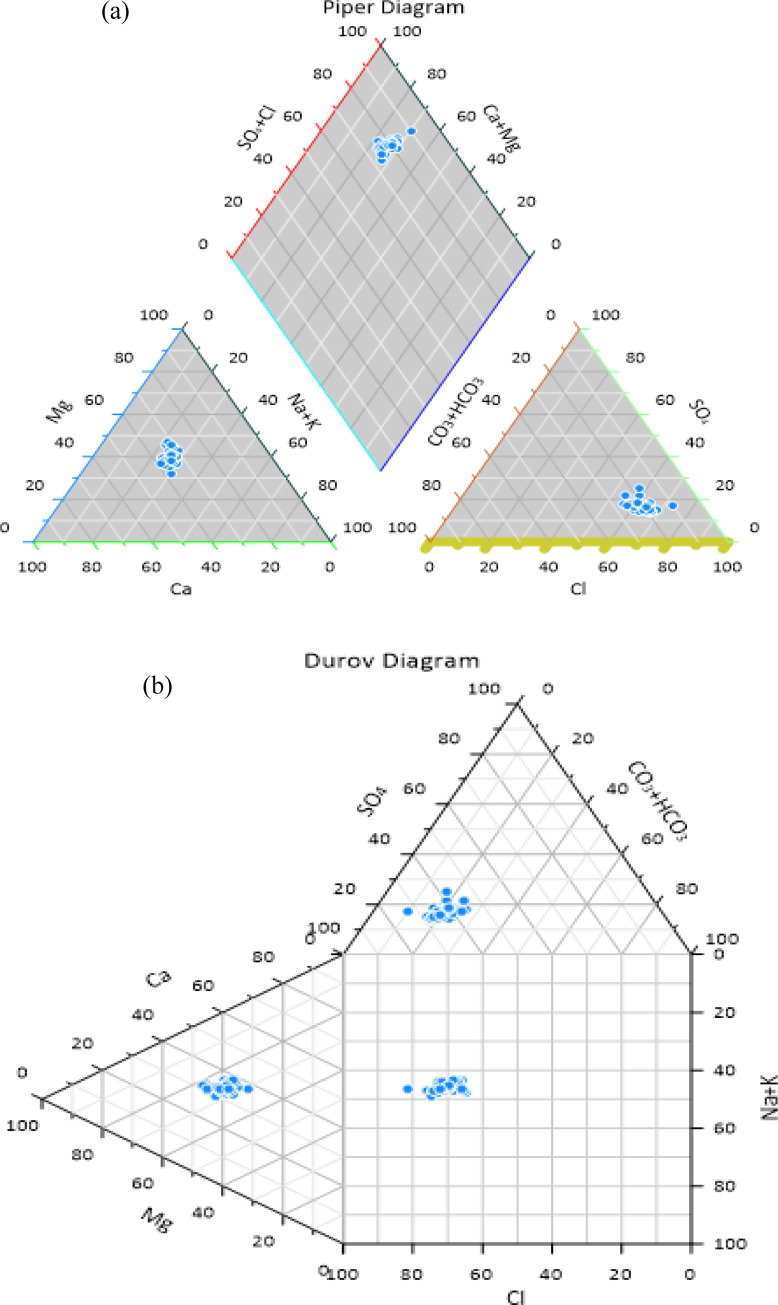
**(a)** Piper diagram and **(b)** Durov diagram.

The Piper Trilinear Diagram [59] and the Durov Diagram [60] were utilized to categorize the water class and formations based on the major ions present. To construct these diagrams, Grapher software was employed. Initially, a text file containing the concentrations of anions and cations was prepared. This file was then uploaded into Grapher, where all the data was transferred to an Excel sheet within the software. Subsequently, using this plotted data, both the Piper and Durov diagrams were drawn. Similarly, the Wilcox Diagram, which plots Electrical Conductivity (EC) against Sodium Percentage (Na%), was created using a separate software designed for diagram drawing. This involved taking a text file with the concentrations of cations and anions, separated by commas, uploading it, and then drawing the Wilcox curve to assess the river water quality for irrigation purposes [61, 62]. The analysis revealed, as shown in Piper’s trilinear diagram (Fig 4A) and Durov diagram (Fig 4B), that the coastal river water in the deltaic region of Bangladesh predominantly falls into the NaCl and NaHCO_3_ categories, with sodium (Na^+^) as the dominant cation and chloride (Cl^-^) and bicarbonate (HCO_3_^-^) as the prevalent anions in most river water samples [61].

Multivariate statistical analyses were conducted using IBM SPSS version 23 for Windows, including frequency distribution (FD) and Q-Q plots, Pearson’s correlation coefficient (PCC), principal component analysis (PCA), and hierarchical cluster analysis (HCA). These analyses were essential to provide a multivariate perspective on the relationships between the calculated parameters. Reliability analysis was employed to quantify the scale of relationships between individual items and their reliability. The internal consistency and group relatedness were estimated using Cronbach’s alpha test. Cronbach’s alpha reliability coefficients are classified as follows: exceptional (>0.90), good (>0.80), acceptable (>0.70), questionable (>0.60), poor (>0.50), and unacceptable (≤0.50). Cronbach’s alpha values above 0.81 indicate reliable data and strong correlations among water quality metrics for the analyzed samples. Table 2 lists all Cronbach’s alpha values. The suitability of data for PCA or factor analysis was evaluated using the Kaiser-Meyer-Olkin (KMO) test and Bartlett’s test of sphericity. The KMO test measures the adequacy of sampling for each variable in the model. A KMO value close to 1.0 suggests high suitability for factor analysis, while a value around 0.50 indicates unsuitability. Values between 0.8 and 0.9 are considered exceptional, 0.7 to 0.8 are good, 0.5 to 0.7 are mediocre, and values above 0.5 are deemed acceptable. In this study, the KMO value was 0.83, indicating that the sampling method is statistically significant for factor analysis. Bartlett’s test of sphericity assesses the appropriateness of the data for factor analysis. Table 2 presents the results of Bartlett’s test (values less than 0.05). The water samples showed a chi-square (χ2) value of 948.99 with 171 degrees of freedom (D_f_). The factor analysis is considered valid since the p-value (Sig.) is 0.000, which is less than 0.05.

## 3. Results and discussion

### 3.1. General status of river water

The results of the water quality parameters from river water samples are summarized in Table 3. The measured values ranged as follows: pH from 6.89 to 8.0 (mean: 7.38), dissolved oxygen from 4.5 to 8.2 mg/L (mean: 6.34 mg/L), electrical conductivity from 1.78 to 2.7 mS/cm (mean: 2.12 mS/cm), carbon dioxide from 8.8 to 15.0 mg/L (mean: 11.92 mg/L), turbidity from 48 to 130 NTU (mean: 98.36 NTU), water temperature from 27 to 32°C (mean: 29.53°C), total dissolved solids (TDS) from 832 to 1350 mg/L (mean: 1106.13 mg/L), total hardness from 126 to 397 mg/L (mean: 287.27 mg/L), and salinity from 1.08 to 2.30 ppt (mean: 1.72 ppt) respectively. The concentrations of dissolved metal ions Na^+^, K^+^, Fe^2+^, Ca^2+^, and Mg^2+^ in the river water samples were observed to be 86–162 mg/L (mean: 110.36 mg/L), 12–62 mg/L (mean: 35.0 mg/L), 0.05–1.05 mg/L (mean: 0.29 mg/L), 37–69 mg/L (mean: 55.96 mg/L), and 15–48 mg/L (mean: 30.02 mg/L), respectively. The minimum value of Na^+^ (86 mg/L) was detected at the Nijum dwip (S36), while the maximum value of Na^+^ (162 mg/L) was found at New Moor (S15). Among the investigated sample results, the Na^+^ cation was dominant in river water. The concentrations of anions such as Cl^-^, NO_3_^-^, HCO_3_^-^, PO_4_^3-^ and SO_4_^2-^ in river water samples are found in the ranges of 106.0–389.0 mg/L (mean: 284.22 mg/L), 3.05–12.0 mg/L (mean: 7.22 mg/L), 76.9–242.2 mg/L (mean: 175.2 mg/L), 0.32–1.3 mg/L (mean: 0.95 mg/L) and 200–421 mg/L (mean: 283.16 mg/L), respectively. These results revealed that the prime anions in the investigated samples were HCO_3_^-^ and Cl^-^. The maximum concentrations of HCO_3_^-^ and Cl^-^ (242.2 mg/L and 284.2 mg/L) were found at Coxbazar and Dublar Char, while the minimum concentrations of HCO_3_^-^ and Cl^-^ (76.9mg/L and 106 mg/L) were also found at Teknaf and Sandwip (S17 and S24).

**Table 3:** Assessed parameters in river water with their descriptive statistics and the entropy water quality index (EWQI) calculation.

Parameters	Water Temp	Salinity (%)	pH	DO (mg/L)	CO_2_ (mg/L)	Turbidity (NTU)	HCO_3_^-^ (mg/L)	EC (mS/cm)	TDS (mg/L)	Hardness (mg/L)	NO_3_^-^ (mg/L)	Cl^-^(mg/L)	SO_4_^2-^ (mg/L)	PO_4_^3-^(mg/L)	Na (mg/L)	K (mg/L)	Ca (mg/L)	Mg (mg/L)	Fe (mg/L)
S1	32	2.1	7.5	6.5	11	80	193.4	2.6	1312	317.1	5.1	310	320	1.2	138	20	55	35	1.03
S2	31	2.0	7.95	6.6	14	85	192.8	2.7	1350	316	5.8	340	343	1.0	143	24	60	38	0.56
S3	30	2.3	7.92	6.7	12.6	60	197.6	2.4	1235	324	4.9	210	302	1.1	128	32	62	38	0.73
S4	29	1.8	7.85	5.9	13.6	114	223.3	2.65	1338	366	5.4	330	345	1.04	132	21	58	44	0.22
S5	30	1.7	7.1	6.0	11.1	115	201.0	2.0	1040	329.5	10	334	200	0.95	102	30	65	42	0.1
S6	30	1.7	7.1	6.0	11.2	116	189.7	1.95	1108	311	11	375	207	0.98	101	23	63	34	0.2
S7	29	1.8	7.2	6.0	11.3	121	227.8	2.1	1020	373.5	10	325	226	0.89	109	34	58	33	0.12
S8	31	1.8	7.1	6.0	11.1	120	192.5	2.1	1010	315.5	9.9	178	215	1.3	102	38	66	41	0.27
S9	31	1.6	7.2	6.5	11.2	125	198.3	2.0	1224	325	8.9	374	221	1.1	102	60	63	40	0.1
S10	30	1.7	7.25	6.6	11.3	121	190.3	2.0	1170	312	8.09	346	290	1.02	109	25	60	36	0.23
S11	30	1.9	7.3	7.8	12.1	110	223.6	1.98	1180	366.5	9.89	354	262	1.06	90	62	63	38	0.14
S12	29	1.8	7.28	8.1	12.1	124	225.1	1.95	1050	369	8.07	302	236	1.11	103	52	69	41	0.25
S13	29	1.7	7.9	8.2	14.2	130	189.7	2.4	1220	311	8.9	389	228	1.09	151	72	64	35	0.16
S14	31	1.7	7.8	7.0	14.1	120	218.1	2.4	1231	357.5	9.96	343	265	1.06	109	67	67	36	0.15
S15	30	1.7	8.0	7.0	15	110	190.6	2.6	1324	312.5	12	328	290	1.06	162	87	65	33	0.12
S16	29	1.56	7.05	6.0	10	68	174.8	1.9	1092	286.5	4.2	106	305	0.5	102	12	37	21	0.75
S17	28	1.08	6.9	4.9	9.9	52	164.4	1.97	1090	269.5	3.05	289	312	0.34	102	14	45	22	0.43
S18	29	1.85	6.89	5.67	8.8	48	152.2	1.8	998	249.5	3.1	278	290	0.32	107	18	47	27	0.43
S19	28	1.32	6.95	5.07	9.95	75	120.2	2.0	1010	197	3.23	197	323	1.01	117	16	40	21	0.09
S20	29	1.35	7.0	5.98	10.87	80	167.8	1.9	898	275	4.3	186	245	0.56	112	22	38	28	0.05
S21	29	1.37	7.05	4.97	11	86	149.8	1.91	980	245.5	4.67	170	311	0.76	102	17	53	22	0.09
S22	28	1.43	7.04	5.93	11.13	85	125.1	1.89	870	205	4.87	178	312	0.69	102	26	43	19	0.08
S23	30	1.54	7.05	4.5	11.4	83	96.1	1.78	879	157.5	4.94	189	205	1.1	101	24	47	17	0.14
S24	30	1.12	6.93	5.97	8.8	82	100.0	1.8	898	164	4.97	129	216	0.9	102	32	42	16	0.05
S25	29	1.24	7.1	6.67	11.26	83	113.5	1.78	832	186	5.02	147	211	0.8	105	34	39	15	0.12
S26	29	1.47	7.1	6.8	11.07	90	111.6	1.91	865	183	4.79	278	277	0.86	90	41	49	18	0.08
S27	28	1.32	7.13	5.7	11.2	78	98.2	1.9	986	161	4.89	249	234	0.78	102	35	48	17	0.19
S28	28	1.21	7.13	6.3	11.2	81	77.5	2.0	1023	127	4.98	187	324	0.98	103	54	46	20	0.21
S29	27	1.3	7.32	6.23	11.4	79	81.1	2.0	1067	133	4.79	276	345	0.89	99	35	43	16	0.18
S30	28	1.54	7.65	5.89	12.5	92	76.9	2.04	1098	126	5.23	220	367	0.98	124	54	41	17	0.16
S31	31	2.2	7.69	6.3	12.5	78	226.9	2.2	1279	372	5.4	309	380	1.02	121	47	52	25	1.05
S32	30	2.1	7.09	6.2	11.02	82	208.9	2.6	1310	342.5	5.6	326	421	1.05	125	49	56	23	0.66
S33	30	2.2	7.85	6.3	13.1	63	182.7	2.45	1238	299.5	5.1	379	401	1.01	128	22	60	31	0.7
S34	30	1.9	7.39	6.1	12.2	110	183.9	2.61	1321	301.5	5.16	323	405	1.04	107	21	52	30	0.45
S35	30	1.9	7.95	6.5	14.2	112	239.4	2.05	1038	392.5	10.09	327	201	0.9	105	26	61	23	0.34
S36	30	1.95	7.09	6.2	11.4	111	157.7	1.98	1074	258.5	11.12	328	232	0.92	86	24	61	24	0.4
S37	28	1.85	7.54	6.1	12.6	114	150.4	2.01	1001	246.5	10.26	356	216	0.88	127	34	63	32	0.16
S38	30	1.85	7.34	6.1	11.3	113	152.8	2.05	1059	250.5	9.67	273	223	1.06	98	24	67	34	0.32
S39	30	1.8	7.05	6.35	11.3	114	242.2	1.97	1088	397	9.23	339	227	1.07	105	32	62	36	0.24
S40	29	1.9	7.89	6.25	13.4	118	237.9	1.98	1057	390	8.9	346	243	1.08	101	32	64	32	0.32
S41	29	1.95	7.45	7.15	11.8	117	226.9	1.92	994	372	9.99	179	237	1.05	95	21	62	38	0.18
S42	30	1.85	7.56	7.5	12.1	120	207.7	2.13	1152	340.5	8.79	301	313	1.07	108	36	68	36	0.3
S43	30	1.9	7.87	7.55	14.4	125	212.6	2.45	1284	348.5	8.99	359	317	1.05	104	52	69	40	0.21
S44	30	1.95	7.75	6.86	14.1	122	194.0	2.45	1205	318	9.89	349	332	1.02	102	48	62	48	0.21
S45	31	2.1	7.9	6.8	14.79	114	198.9	2.23	1278	326	11.9	379	367	1.06	103	61	63	39	0.33
Mean	29.53	1.72	7.38	6.35	11.92	98.36	175.2	2.12	1106.1	287.27	7.22	284.22	283.16	0.95	110.36	35	55.96	30.02	0.3
SD	1.06	0.3	0.36	0.76	1.5	22.37	47.6	0.27	145.05	78.11	2.7	77.98	62.07	0.2	16.03	5.9	9.7	9.1	0.24
Sum	1329	77.4	332.2	285.74	536.59	4426	7885.5	95.49	49776	12927.1	325.03	12790	12742	42.71	4966	36.33	2518	1351	13.3
Min	27	1.08	6.89	4.5	8.8	48	76.9	1.78	832	126	3.05	106	200	0.32	86	12	37	15	0.05
Max	32	2.3	8	8.2	15.0	130	242.2	2.7	1350	397	12	389	421	1.3	162	87	69	48	1.05
Ej			1	0.95	0.85	0.99	0.98	0.97	0.95	0.95	0.97	0.99	0.98	1.01	1	0.87	0.96	0.97	0.95
Wj			0	0.05	0.05	0.01	0.01	0.03	0.05	0.05	0.03	0.01	0.02	-0.01	0	0	0.04	0.03	0.02
Sj			7	6	-	20	600	250	300	500	-	250	200	-	200	30	75	35	0.3
Qj			0	5.92	0	9.8	0.92	0.03	18.44	2.87	0	1.14	2.83	0	0	0	2.98	2.57	2.0
EWQI	49.56																			

### 3.2. Quality assessment of investigated water

Various organizations, including the Bureau of Indian Standards (BIS) in 2012, the U.S. Environmental Protection Agency (US EPA) in 2018, the World Health Organization (WHO) in 2011, and The Environment Conservation Rules (ECR) in 1997, have established standard permissible limits for drinking water quality parameters. No sampling sites exceeded the standard limit for sodium concentrations, which is set at 200 mg/L. Furthermore, all sample sites, S1 through S45, exceeded the recommended chloride concentration range of 150–250 mg/L. During the pre-monsoon and post-monsoon seasons, the bicarbonate levels at 67% of the sampling sites (30 sites), specifically S1-S15 and S31-S45, surpassed the recommended values. The entropy water quality index (EWQI) was utilized to assess the quality of river water by comparing the significance of each analytical parameter [58]. According to the BIS (2012), US EPA (2018), WHO (2011), and ECR (1997) guidelines, the EWQI values for each of the river water quality parameters, such as pH, DO, EC, TDS, total hardness, Na^+^, K^+^, Fe^2+^, Ca^2+^, Mg^2+^, Cl^-^, SO_4_^2-^, and HCO_3_^-,^ were calculated.

**Fig 5:**
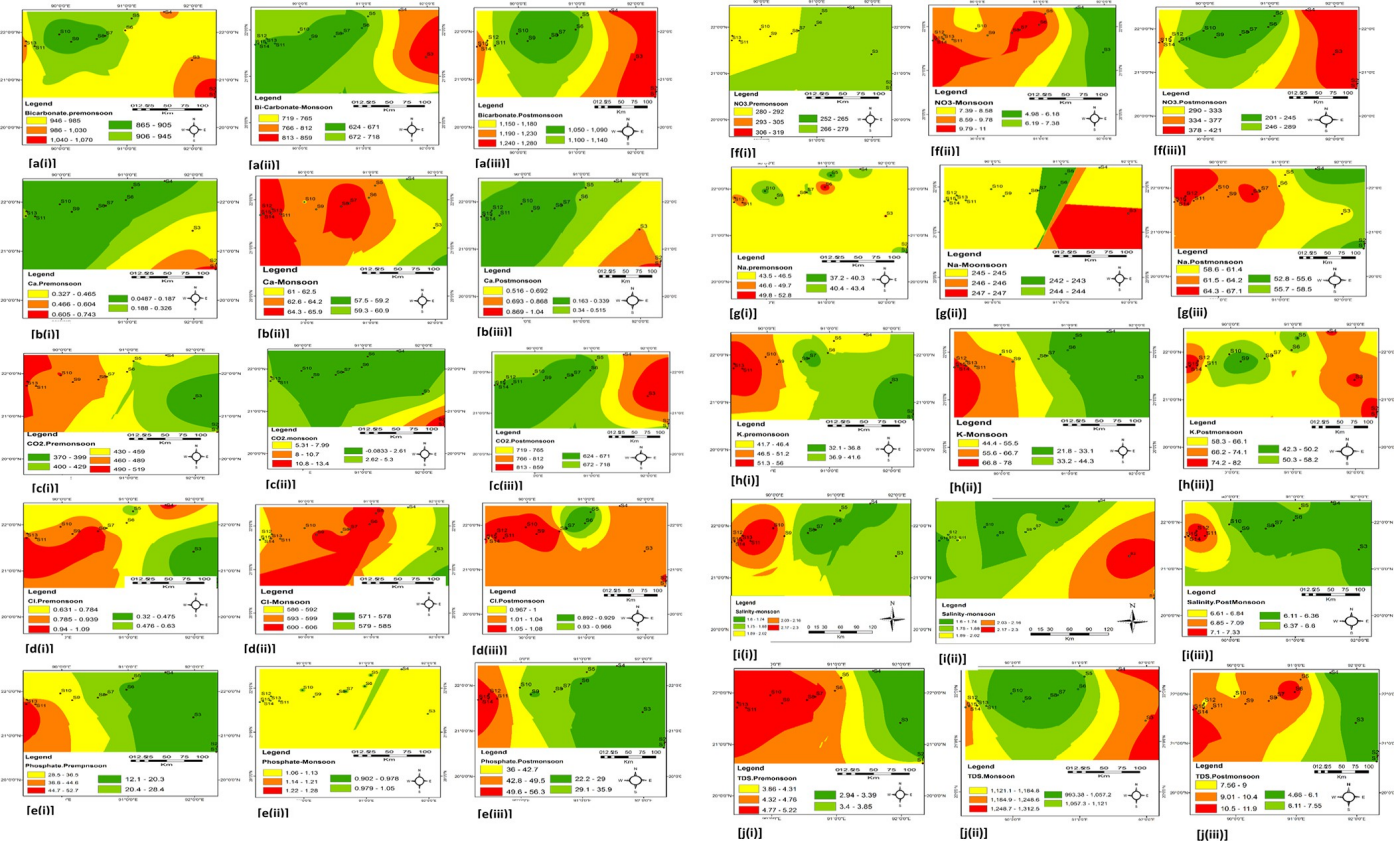
a-j (i, ii, iii) The spatial distribution of the three seasons and their concentrations of HCO_3_^-^ Ca^2+^, CO_2_, Cl^-^, PO_4_^3-^, NO_3_^-^, Na^+^, K^+^, salinity, and TDS.

The spatial distribution maps, as shown in Fig 5 [a-j (i, ii, iii)] and the Supplementary map in S1 Fig [k-s (i, ii, iii)], were created to illustrate the concentrations of these parameters and evaluate seasonal variations.

Water samples can be categorized into five groups based on the EWQI values, and they are extremely poor (unacceptable, EWQI ≥ 200), poor (not suitable for drinking, 150 ≤ EWQI < 200), industrial usage, 100 ≤ EWQI < 150), moderate (for domestic, irrigation), suitable for drinking (50 ≤ EWQI <100) and excellent for drinking (EWQI <50). The EWQI value was found in the range between 0.013 and 7.026 with a mean value of 20.87, indicating that the investigated water samples are suitable for drinking (EWQI*>*50). It also illustrates a homogeneous distribution of water quality for all sampling sites. Evaluated parameters usually influenced the water quality with minimal entropy (ej) and entropy weightage (ωj) values [57]. Depending on the EWQI values shown in Table 3, the evaluated water quality parameters followed the order of TDS >Turbidity > DO> Hardness > SO_4_^2-^> Ca^2+^ > Mg^2+^ > Fe^2+^ > Salinity> Cl^-^>HCO_3_^-^> EC> Na^+^> K^+^ > pH. Based on the EWQI values, water quality from diverse sampling stations disintegrated as S10 > S11 *>* S40 > S8 > S15 *>* S6 *> S36>* S45 *>S13 >* S2 *>* S38> S4 > S3 > S42 > S1 > S41 > S32 > S34 > S43 > S12 > S9 > S37 > S7 > S33 > S31 > S44 > S14 > S39 > S35 > S5. The Na^+^ concentration percentage in the analyzed samples was determined to assess their suitability for agricultural use [62]. The observed concentrations of Na^+^ and HCO_3_^-^ suggest the presence of alkaline water, which may originate from lithological rock, coastal aquifers, and seawater. When mixed with Cl^-^ ions, this water becomes saline. For water to be suitable for agricultural purposes, the maximum allowable Na^+^ concentration should not exceed 15% [48, 62]. According to the guidelines set by Wilcox (1948) [62] and Roy et al. (2019) [61], most of the water samples analyzed fell into the doubtful category regarding suitability for irrigation (as depicted in Fig 5), with only two samples classified as unsuitable.

### 3.3. Pearson’s correlation coefficient

**Fig 6:**
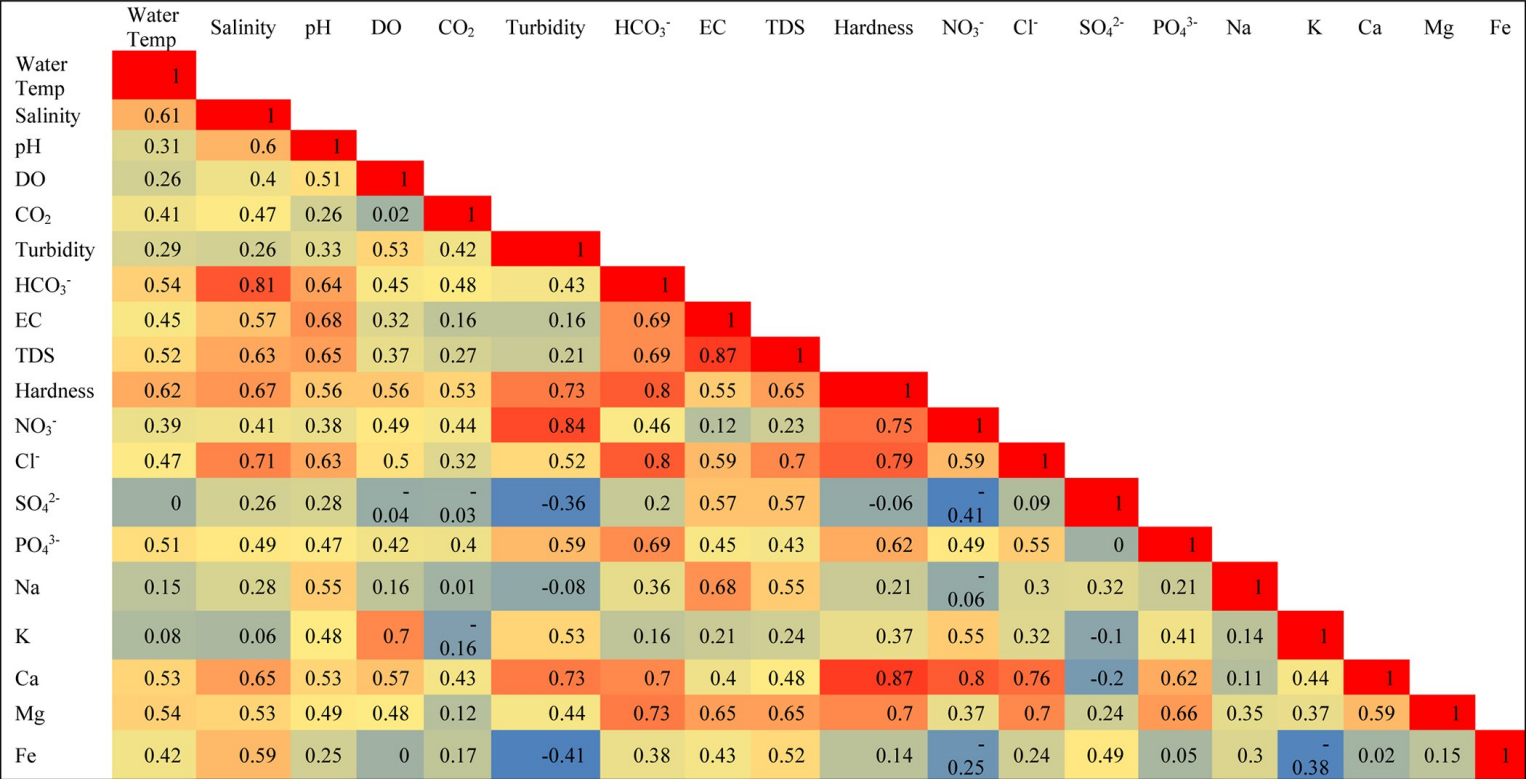
Heat map of Pearson’s correlation matrix of the parameters for water samples.

Pearson’s correlation was utilized to assess the relationships between parameters in the samples. Pearson’s correlation coefficients (r) are considered significant at the 95% confidence level for the t-test and at the 99% confidence level for the alpha test [63, 64]. The correlation coefficient (r) was classified as "very strong" (0.80–1.00), "strong" (0.60–0.79), "moderate" (0.40–0.59), "weak" (0.20–0.39), and "extremely weak" (0.00–0.19). Fig 6 demonstrates both negative and positive correlations among quality parameters and computed variables. A positive correlation indicates that parameters sharing common sources are not significantly influenced by additional sources, reflecting similar environmental behaviours. Conversely, a negative correlation suggests that the spatial distribution of parameters is affected by common causes, yet their environmental behaviors differ. For instance, electrical conductivity (EC) correlates with total dissolved solids (TDS), salinity, pH, bicarbonate, Na, chloride, sulfate, and Mg; chloride correlates with salinity, pH, dissolved oxygen (DO), turbidity, bicarbonate, EC, phosphate, hardness, nitrate, Ca, and Mg; bicarbonate correlates with water temperature, salinity, pH, hardness, phosphate, EC, chloride, Ca, and Mg; and Na correlates with EC and TDS. Strong positive correlations indicate a common origin and similar behavior in the examined environment, while moderate positive correlations suggest a slightly different origin. The relationships between related water quality metrics ranged from very weak to weak, suggesting a modest contribution to overall water quality.

### 3.4. Principal component analysis (PCA)

Principal component analysis (PCA) is a powerful technique for identifying similarities within a dataset. Data transformation methods like PCA can uncover any underlying patterns in a multivariate dataset [65]. According to the Kaiser criterion, eigenvalues greater than 1 are considered significant and retained, determining the number of principal components (PCs) to be kept [65].

**Fig 7:**
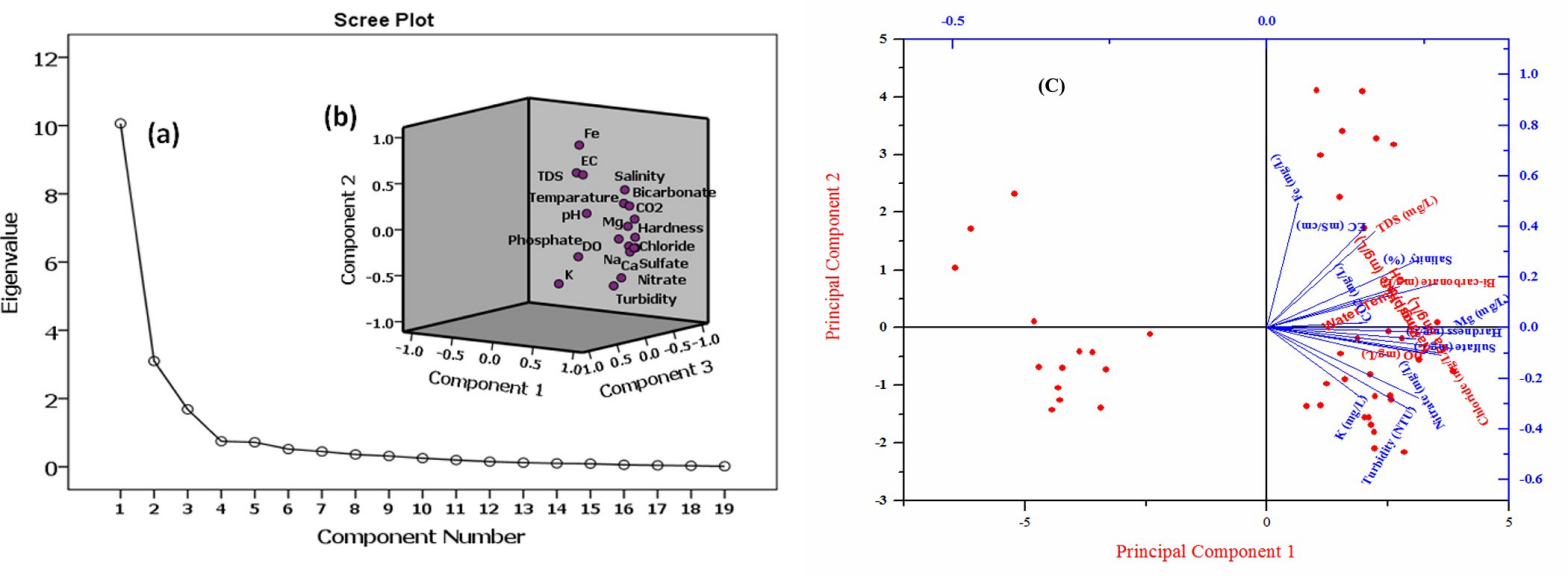
Principal component analysis of **(a)** cumulative % of loading factor for eigenvalues and component number, **(b)** scree plot of the eigenvalues and rotated space, and **(c)** PC1 versus PC2.

This criterion is used to maximize the variance explained by the PCs. Table 4 and Fig 7 detail the loadings of the PCs and their respective variances for the water samples analyzed. Loadings with significance values greater than 0.7 are highlighted to indicate their importance. The aim of rotation in PCA is achieved by revealing many near-zero loadings and a few strong loadings, which clearly distinguish each component. To maximize the variance explained by the principal components (PCs), an effort is made to achieve loadings that are extremely high (positive or negative) or nearly zero, as noted by Davis (1986). For the water samples in this study, all three identified components have eigenvalues greater than 1, as detailed in Table 4 (10.1, 3.1, and 1.7). The scree plot (Fig 7) demonstrates that these three PCs account for 78.3% of the total variance in the data set under investigation. High eigenvalues signify the importance of each component in representing the data, with the total variances explained by PC1, PC2, and PC3 being 52.95%, 16.3%, and 8.76%, respectively, for water quality data. According to the loading values of 0.94, 0.87, 0.94, 0.86, 0.81, 0.88, and 0.83, with an eigenvalue of 10.1, elements such as hardness, chloride, bicarbonate, sodium, calcium, and magnesium are strongly and positively correlated with PC1. This suggests a significant association of these elements in the water, likely due to the dissolution of calcite or dolomite. PC2, with an eigenvalue of 3.1 and a loading value of 0.87, is strongly associated with iron (Fe) alone, indicating its distinctive influence on the water quality. PC3 does not exhibit significant correlations with any variables, highlighting the unique contributions of PC1 and PC2 to the water quality analysis. These findings underscore the significance of these components in classifying the water quality and suggest saltwater intrusion into the coastal aquifer at the studied site, as indicated by these quality parameters.

**Table 4:** Principal component loadings and explained variances in water samples.

	Principal Component Analysis			
Parameters (R-mode)	PC1	PC2	PC3	Communalities
Water Temp	0.65	0.26	-0.27	0.57
Salinity (%)	0.76	0.44	-0.14	0.79
pH	0.68	0.26	0.427	0.72
DO (mg/L)	0.63	-0.21	0.49	0.68
CO2 (mg/L)	0.53	0.029	-0.63	0.69
Turbidity (NTU)	0.71	-0.59	-0.02	0.85
TDS (mg/L)	0.58	0.66	0.34	0.88
EC (mS/cm)	0.52	0.68	0.37	0.87
Hardness (mg/L)	0.94	-0.05	-0.07	0.89
Nitrate (mg/L)	0.75	-0.51	-0.09	0.83
Chloride (mg/L)	0.87	-0.18	-0.18	0.82
Sulfate (mg/L)	0.94	-0.16	-0.04	0.92
Phosphate (mg/L)	0.76	-0.08	-0.03	0.59
Bi-carbonate (mg/L)	0.86	0.29	-0.07	0.83
Na (mg/L)	0.81	-0.16	-0.13	0.71
K (mg/L)	0.49	-0.5	0.64	0.88
Ca (mg/L)	0.88	-0.21	-0.06	0.82
Mg (mg/L)	0.83	0.06	-0.1	0.7
Fe (mg/L)	0.19	0.87	-0.14	0.82
Eigenvalues	10.1	3.1	1.7	
% of variance	52.95	16.3	8.85	
Cumulative %	52.95	69.25	78.01	

The PCA value illustrates how seawater intrusion mostly pollutes the water quality at the deltaic site.

**Fig 8:**
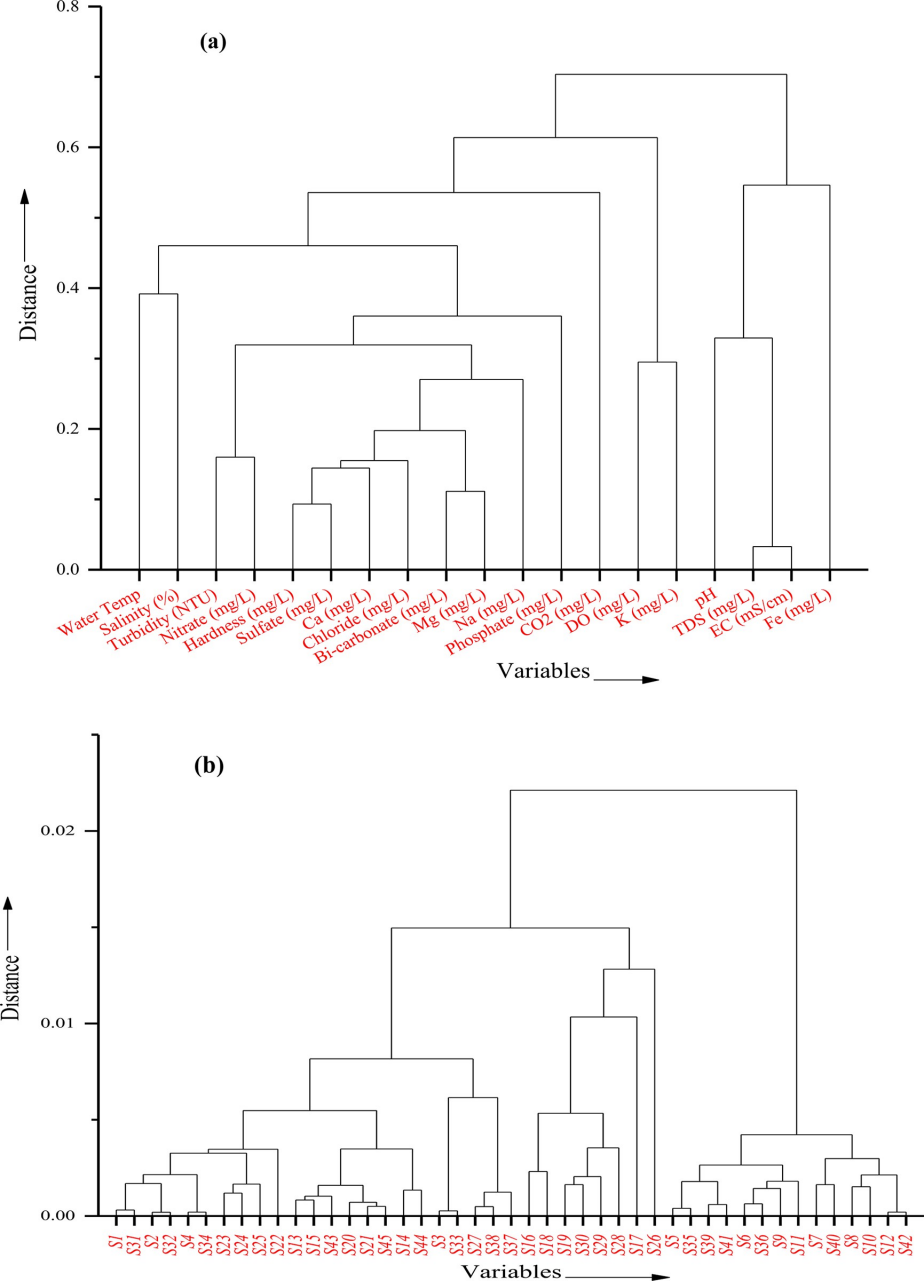
The dendrogram was obtained from hierarchical clustering analysis for **(a)** assessed water quality parameters (distance vs. variables) and **(b)** sampling sites (distance vs variables).

Principal Component Analysis (PCA) and Hierarchical Cluster Analysis (HCA) were employed to identify and categorize spatial similarities among water samples according to their analogous behavior with respect to evaluated water quality metrics [66]. The dendrogram resulting from HCA, which utilizes Ward’s method and a measure of similarity for evaluation, can be segmented into three main clusters (see Fig 8A). The contaminants of the seawater source indicated in PC1 and Table 4 as Cluster 1 included the water temperature, total hardness, bicarbonate, turbidity, CO_2_, Mg^2+^, Ca^2+^, PO_4_^3-^, SO_4_^2-^, HCO_3_^-^, salinity, Na^+^, Cl^-^, and NO_3_^-^. Cluster 2, which was found in PC2 (Table 4) and comprised DO and K^+^, suggesting that dissolved oxygen and potassium minerals were mixed in the river water. Cluster 3 of the pH, TDS, E.C., and Fe^2+^ indicators shows that iron ore is leaching into the water body. A statistically significant correlation was found among the evaluated parameters in the analyzed water samples. This correlation mirrored the similarities between the variance explained by Principal Component Analysis (PCA) and the variance within the clustering of sampling locations as determined by Cluster Analysis (CA), as illustrated in Fig 8B. The concordance between the PCA and CA results supports the connection among inherent water quality parameters. The observed common and significant positive correlation among these parameters, given their sources of origin, is likely due to geogenic activities, seawater intrusion, and the dissolution of carbonate minerals. Hierarchical Cluster Analysis (HCA) grouped the sampling sites into three clusters based on their similarities: HCA1 comprised sites S1, S31, S2, S32, S4, S34, S23, S24, S25, S22, S13, S15, S45, S20, S21, S43, S14, S44, S3, S33, S27, S37, and S38; HCA2 included sites S16, S18, S19, S30, S29, S28, S17, and S26; HCA3 consisted of sites S5, S35, S39, S36, S6, S41, S9, S11, S7, S40, S8, S10, S12, and S42.

The sodium adsorption ratio (SAR) significantly influences the water infiltration rate, making a low SAR preferable for maintaining soil health. The percentage of sodium (%Na) is critical in evaluating the suitability of groundwater for irrigation purposes because high concentrations of sodium ions in irrigation water can interact with soil particles, reducing their permeability [68]. When water contains a high Na^+^ concentration, clay minerals in the soil absorb these ions and release Ca^2+^ and Mg^2+^ ions. This exchange of Na^+^ in the water for Ca^2+^ and Mg^2+^ in the soil reduces permeability and, consequently, soil infiltration rates. As a result, soils often become compact and hard during dry conditions, while the movement of air and water through the soil is restricted under wet conditions [69]. The SAR index is a measure of the soil’s ability to exchange Ca^2+^ and Mg^2+^ ions for Na^+^ ions in groundwater at ion-exchangeable sites. This exchange leads to soil particle dispersion and a decrease in infiltration capacity [70]. Despite potential issues with high salinity, irrigation water can still be beneficial for the land. The Kelly’s Index (KI) was calculated to determine whether groundwater could be utilized for irrigation purposes [71].

**Fig 9:**
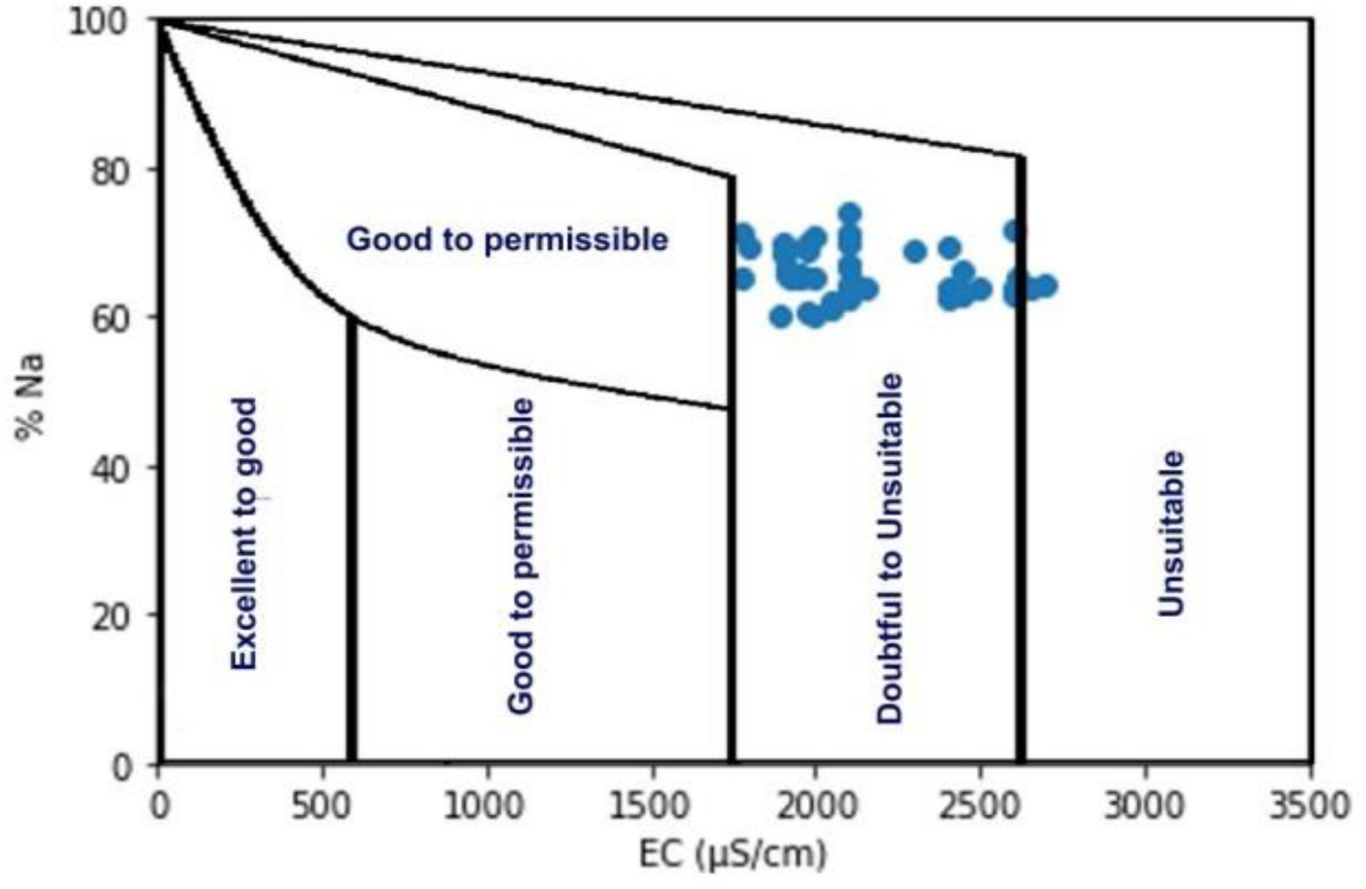
Classification of river water samples for irrigation purposes (after Wilcox 1948) in the coastal belt, Bangladesh.

SAR values ranged between 16.87 and 21.18 mg/L, with a mean value of 16.83 mg/L. The SAR values for the pre-monsoon, monsoon, and post-monsoon seasons were 16.86, 18.63, and 15.80, respectively, as listed in Table 5. The SAR values indicate that the water quality is not alarming across all samples. However, according to the Wilcox diagram [49], the water samples from the study area are classified as unsuitable for irrigation purposes (Fig 9). Wilcox [49] also provided a classification for irrigation water based on the percentage of sodium (%Na), as depicted in Fig 9. The mean soluble sodium percentage (%Na) values for pre-monsoon, monsoon, and post-monsoon were 63.0 mg/L, 61.0 mg/L, 67.0 mg/L, and 60.0 mg/L, respectively. Additionally, the mean residual sodium carbonate (RSC) values for the pre-monsoon, monsoon, and post-monsoon seasons were found to be 89.22 mg/L, 103.46 mg/L, 57.01 mg/L, and 107.31 mg/L, respectively. These results suggest that the water is generally unsuitable for irrigation.

**Table 5:** Agriculture suitability evaluation [67].

Index	Range	Water Category	Mean	Pre-monsoon	Monsoon	Post-monsoon
%Na	20–60	Permissible	63.0	61.0	67.0	60.0
	20–40	Good				
	<20	Excellent				
SAR	>26	Unsuitable	16.83	16.86	18.63	15.80
	18–26	Doubtful or fairly poor				
	10–18	Good				
	<10	Excellent				
MAR	>60	Unsafe	34.92	37.53	30.94	34.43
	<60	Safe				
KR	>1	Unsuitable	1.28	1.19	1.66	1.16
	<1	Good				
PI	<25%	Unsuitable—Class III	145.44	147.27	133.88	153.7
	25–75%	Good—Class II				
	>75%	Good—ClassI				
RSC	>2.5	Unsuitable	89.22	103.46	57.01	107.31
	1.25–2.5	Marginal				
	<1.25	Safe				

Excessive carbonates and bicarbonates, relative to calcium (Ca^2+^) and magnesium (Mg^2+^) ions, can compromise the quality of irrigation water by causing the precipitation of these alkali earth metals, particularly Ca^2+^ and Mg^2+^. This precipitation of Ca^2+^ and Mg^2+^ as carbonate minerals may lead to an increase in both the sodium adsorption ratio (SAR) and the concentration of sodium ions [72]. Furthermore, the high residual sodium carbonate (RSC) can result in the dissociation of organic matter, which ultimately leaves a black residue on the soil’s surface upon drying. This phenomenon has the potential to deteriorate the soil’s physical properties [73, 74]. The mean Magnesium Adsorption Ratio (MAR) values for pre-monsoon, monsoon, and post-monsoon were 34.92 mg/L, 37.53 mg/L, 30.94 mg/L, and 34.43 mg/L, respectively. According to Table 5, the water is considered safe for irrigation as the MAR values are below 60 mg/L. Conversely, the values for Kelly’s Ratio (KR) were found to be 1.28, 1.19, 1.66, and 1.16, respectively, indicating that the water is unsuitable for irrigation when the KR is greater than 1. Furthermore, the Permeability Index (PI) values were 144.45 mg/L, 147.27 mg/L, 133.88 mg/L and 153.7 mg/L, respectively. These PI values categorize the water as Class III, indicating it is unsuitable for irrigation purposes.

## 4. Conclusion and recommendations

River water is an essential source of freshwater, crucial for supporting aquatic life, irrigation, industrial uses, environmental conservation, and drinking. Agricultural projects and natural disasters, such as hurricanes, can affect the freshwater availability in downstream areas. Consequently, salinity intrusion has been progressively affecting river water in Bangladesh, especially across the coastal belt. Typically, the salinity level of freshwater is less than 0.5 parts per thousand (ppt), but this study found that salinity levels in the coastal belt of Bangladesh ranged from 1.7 to 2.3 ppt. River salinity was observed to be lower during the monsoon season and higher during the dry season, which spans from March to April. Overall, the water quality in the coastal belt of Bangladesh has not exceeded the permissible values for general use. However, the Entropy Water Quality Index (EWQI) and Sodium Adsorption Ratio (SAR) data suggest that the samples analyzed are suitable for drinking but unsuitable for irrigation. Additionally, the Permeability Index (PI), Kelly’s Ratio (KR), and Residual Sodium Carbonate (RSC) values further indicate that the water is unsuitable for irrigation or farming purposes.

To mitigate salinity intrusion in the coastal surface waters, sustainable polder development initiatives should be launched, including the construction of twister technology or cement concrete block (CC block) dams, routine dredging of river sediments, and halting the construction of upstream dams. To address these activities by neighboring countries, a strategic foreign policy and diplomatic engagement are essential to foster a win-win situation. Dialogues should be initiated in international forums such as the UN, ASEAN, NAM, and SAARC to secure water rights and justice and to combat salinity intrusion in the Teesta, Padma, and Jamuna rivers. Rainwater harvesting can serve as an alternative water source or a viable technique to alleviate salinity intrusion across the coastal belt during the rainy season. The use of groundwater for irrigation and shrimp cultivation should be discontinued. Balancing the upstream discharge rate through projects like the China Teesta Barrage is crucial. Introduction of salinity-tolerant crops through genetic modification or other sustainable methods is recommended. Nature-based solutions, such as planting *Sonneretia apetala* and *Nipa fruticans* trees, should be adopted because these trees not only reduce salinity but also mitigate climate change, protect against natural hazards like cyclones, and lower temperatures. Additionally, their fruits can provide alternative livelihood options for the local community through the production of jam, jelly, pickles, sauce, tea, and more.

## Supporting information

S1 Fig [k-s (i, ii, iii)]: The spatial distribution of three seasons and its concentrations of Hardness, Fe, EC, pH, DO, Mg, Turbidity, SO42- and Water Temperature respectively. (DOCX)
