# Supplementary material for: Assessment of coastal river water quality in Bangladesh: Implications for drinking and irrigation purposes
Source: PLoS One. 2024 Apr 18;19(4):e0300878. doi: 10.1371/journal.pone.0300878 (PMC11025935; doi:10.1371/journal.pone.0300878)
Supplement: S1 Fig — (DOCX) [file pone.0300878.s001.docx]

Fig. S1 [k-s (i, ii, iii)]: The spatial distribution of three seasons and its concentrations of Hardness, Fe, EC, pH, DO, Mg, Turbidity, SO_4_^2-^ and Water Temperature respectively.
